# Rates of Extreme Neonatal Hyperbilirubinemia and Kernicterus in Children and Adherence to National Guidelines for Screening, Diagnosis, and Treatment in Sweden

**DOI:** 10.1001/jamanetworkopen.2019.0858

**Published:** 2019-03-22

**Authors:** Jenny Alkén, Stellan Håkansson, Cecilia Ekéus, Pelle Gustafson, Mikael Norman

**Affiliations:** 1 Division of Pediatrics, Department of Clinical Science, Intervention and Technology, Karolinska Institutet, Stockholm, Sweden; 2 Department of Neonatal Medicine, Karolinska University Hospital, Stockholm, Sweden; 3 Department of Clinical Science/Pediatrics, Umeå University, Umeå, Sweden; 4 Swedish Neonatal Quality Registry, Umeå, Sweden; 5 Division of Reproductive Health, Department of Women’s and Children’s Health, Karolinska Institutet, Sweden; 6 The Swedish National Patient Insurance, Stockholm, Sweden

## Abstract

**Importance:**

Neonatal hyperbilirubinemia can cause lifelong neurodevelopmental impairment (kernicterus) even in high-resource settings. A better understanding of the incidence and processes leading to kernicterus may help in the design of preventive measures.

**Objectives:**

To determine incidence rates of hazardous hyperbilirubinemia and kernicterus among near-term to term newborns and to evaluate health care professional adherence to best practices.

**Design, Setting, and Participants:**

This population-based nationwide cohort study used prospectively collected data on the highest serum bilirubin level for all infants born alive at 35 weeks’ gestation or longer and admitted to neonatal care at all 46 delivery and 37 neonatal units in Sweden from 2008 to 2016. Medical records for newborns with hazardous hyperbilirubinemia were evaluated for best neonatal practices and for a diagnosis of kernicterus up to 2 years of age. Data analyses were performed between September 2017 and February 2018.

**Exposures:**

Extreme (serum bilirubin levels, 25.0-29.9 mg/dL [425-509 μmol/L]) and hazardous (serum bilirubin levels, ≥30.0 mg/dL [≥510 μmol/L]) neonatal hyperbilirubinemia.

**Main Outcomes and Measures:**

The primary outcome was kernicterus, defined as hazardous neonatal hyperbilirubinemia followed by cerebral palsy, sensorineural hearing loss, gaze paralysis, or neurodevelopmental retardation. Secondary outcomes were health care professional adherence to national guidelines using a predefined protocol with 10 key performance indicators for diagnosis and treatment as well as assessment of whether bilirubin-associated brain damage might have been avoidable.

**Results:**

Among 992 378 live-born infants (958 051 term births and 34 327 near-term births), 494 (320 boys; mean [SD] birth weight, 3505 [527] g) developed extreme hyperbilirubinemia (50 per 100 000 infants), 6.8 per 100 000 infants developed hazardous hyperbilirubinemia, and 1.3 per 100 000 infants developed kernicterus. Among 13 children developing kernicterus, brain injury was assessed as potentially avoidable for 11 children based on the presence of 1 or several of the following possible causes: untimely or lack of predischarge bilirubin screening (n = 6), misinterpretation of bilirubin values (n = 2), untimely or delayed initiation of treatment with intensive phototherapy (n = 1), untimely or no treatment with exchange transfusion (n = 6), or lack of repeated exchange transfusions despite indication (n = 1).

**Conclusions and Relevance:**

Hazardous hyperbilirubinemia in near-term or term newborns still occurs in Sweden and was associated with disabling brain damage in 13 per million births. For most of these cases, health care professional noncompliance with best practices was identified, suggesting that a substantial proportion of these cases might have been avoided.

## Key Points

**Question:**

What is the incidence of hazardous neonatal hyperbilirubinemia, and does an association exist between the quality of neonatal care and kernicterus?

**Findings:**

In this population-based cohort study of 992 378 live-born children in Sweden from 2008 to 2016, 67 newborns were exposed to serum bilirubin levels of 30 mg/dL (510 μmol/L) or higher, of whom 13 developed kernicterus. Root cause analysis indicated that 11 of these 13 kernicterus cases (85%) were potentially avoidable because they were associated with suboptimal screening, diagnosis, or treatment.

**Meaning:**

Kernicterus observed in a high-resource setting was associated with nonadherence to best practice guidelines and thus might have been prevented in a majority of infants.

## Teaser

This population-based nationwide cohort study evaluates the incidence rates of hazardous hyperbilirubinemia and kernicterus among near-term to term infants in Sweden and assesses health care professionals’ adherence to best practices.

## Introduction

Neonatal hyperbilirubinemia is a major cause of lifelong neurodevelopmental impairment.^1^ Given effective tools for risk assessment and prevention^2,3,4^ as well as for diagnosis and treatment,^5,6,7,8^ devastating neonatal hyperbilirubinemia should be a preventable condition. However, kernicterus occurs in high-resource settings despite evidence-based guidelines^5,9^ and under what could be considered optimal conditions.^10,11,12^ Therefore, a better understanding of the processes leading to kernicterus is needed.

There are a few population-based studies (from Denmark, the United Kingdom, the United States, and Canada) of the incidence of extreme neonatal hyperbilirubinemia and kernicterus.^12,13,14,15,16,17^ Previous studies report diagnoses associated with kernicterus, such as hemolytic or nonhemolytic diseases,^11,15^ bilirubin levels,^14,18^ and root cause analysis,^19^ but there is no previous study, to our knowledge, on hyperbilirubinemia resulting in kernicterus using population-based epidemiology to establish incidence rates with analysis of adherence to best practice guidelines.

In 2008, national guidelines for neonatal hyperbilirubinemia were issued in Sweden. At the same time, the Swedish Neonatal Quality (SNQ) register started to collect information on maximum serum bilirubin levels for all infants admitted to neonatal care. Using these sources of information and the Swedish Medical Birth Registry (MBR), the present study aimed to determine incidence rates of extreme and hazardous neonatal hyperbilirubinemia in a population of almost 1 000 000 term and near-term newborns, the risk factors and causes of hazardous neonatal hyperbilirubinemia, and to what extent kernicterus developed. In addition, the present study also aimed to audit the medical records of all newborns with hazardous hyperbilirubinemia for compliance with national standards and to determine to what extent kernicterus might have been avoidable. Finally, we captured information on the proportions of families affected by kernicterus who had applied for and were granted financial compensation from the Swedish Patient Insurance.

## Methods

### Participants

This was a nationwide population-based cohort study. The full population comprised all live-born children delivered near term or at term (≥35 weeks of gestation) in Sweden during the study period from 2008 to 2016. The cohort consisted of all newborns with extreme hyperbilirubinemia, defined as a maximum total serum bilirubin level of 25.0 to 29.9 mg/dL (425-509 μmol/L). A subsample of the cohort consisted of all newborns with hazardous hyperbilirubinemia, defined as serum bilirubin levels of 30 mg/dL (510 μmol/L) or higher. This subsample underwent medical record review using a predefined protocol (presented below) for root cause analysis and assessment of quality of care. The bilirubin levels used for risk assessment and treatment in Sweden since 2008 are presented in the eFigure in the Supplement. Ethical approval for this study was obtained from the Regional Ethics Review Board in Stockholm, Sweden. Patient informed consent was not obtained because a waiver for the need to obtain such consent was granted by the Regional Ethics Review Board. This report follows the Strengthening the Reporting of Observational Studies in Epidemiology (STROBE) reporting guideline for cohort, case-control, and cross-sectional studies, version 4, October/November 2007.

The medical records were reviewed up to a maximum of 2 years of age for all children who had been exposed to bilirubin levels of 30 mg/dL (510 μmol/L) or higher during the neonatal period. Acute bilirubin encephalopathy was defined as hazardous neonatal hyperbilirubinemia combined with symptoms such as apnea, convulsion, lethargy, irritability, hypotonous, hypertonus, feeding difficulties, or hyperthermia. Kernicterus was defined as hazardous neonatal hyperbilirubinemia associated with later documented cerebral palsy, deafness, gaze paralysis, or neurodevelopmental retardation.

### Data Sources

The Swedish MBR was used to assess the number of births in the study period. The MBR also contains *International Classification of Diseases, Tenth Revision* (*ICD-10*) codes for neonatal diagnoses. We used this information to provide personal identification numbers to track medical records for patients registered with a diagnosis of kernicterus (*ICD-10* code P57) in a previous study conducted from 1999 to 2012.^4^

The SNQ contains information on maximum serum bilirubin levels starting in 2008. We used this register to identify all newborns with extreme and hazardous hyperbilirubinemia. Finally, we extracted all files of claims within the Swedish National Patient Insurance database having *ICD-10* codes for neonatal hyperbilirubinemia.

### Medical Record Review

Personal identification numbers retrieved from the MBR and SNQ registers were used to access medical records of hospital databases and county archives. Records for both the mother and infant were scrutinized according to a predefined protocol by 2 of us (J.A. and M.N.).

First, we checked for misclassification of maximum bilirubin level and gestational age. We found that the majority of the infants registered in the Swedish MBR with an *ICD-10* code of P57 (kernicterus) were misclassified; 35 of 42 newborns had been treated for neonatal hyperbilirubinemia, but no newborn had bilirubin levels higher than 25 mg/dL (425 μmol/L) and no infant developed kernicterus. Two infants who had been given a diagnosis of kernicterus in the MBR were born before 35 weeks of gestation, and in 5 cases, the medical records were untraceable or unavailable. There is no information in the MBR beyond the neonatal period. Accordingly, all cases in the MBR were excluded from the present study. Two infants in the SNQ register were also misclassified and excluded (1 with a maximum bilirubin level lower than 25 mg/dL [425 μmol/L] and the other infant with a gestational age <35 weeks).

Second, we listed the perinatal characteristics, risk factors, and diagnoses for infants with hazardous neonatal hyperbilirubinemia. The 18 predefined risk factors were family history of hemolytic disease, maternal country of origin (southeast Asia, which was defined as being born in Pakistan, India, China, or in countries southeast of these 3, or another country of origin), parity (primipara or multipara), maternal overweight or obesity (defined as having a body mass index >25 [calculated as weight in kilograms divided by height in meters squared]), maternal diabetes (pregestational or gestational), maternal blood group O or Rh negative, older sibling treated for neonatal hyperbilirubinemia, suspected or confirmed isoimmunization before birth, gestational age of 35 to 38 weeks, infant male sex, small or large for gestational age, early neonatal weight loss more than 10%, or documented late start of breastfeeding.^4,5^ Underlying or contributing diagnoses and conditions were categorized as hemolytic or nonhemolytic as specified in the medical records. All newborns with hazardous hyperbilirubinemia had been evaluated for isoimmunization. In cases without a diagnosis of isoimmunization, evaluation for other hemolytic diseases (such as glucose-6-phosphate dehydrogenase [G6PD] deficiency), metabolic disease, liver disease, or infection was standard procedure, but the adherence to this recommendation was not studied.

In a third step, we identified 10 key performance indicators from national^20^ and international^5^ guidelines, of which 7 were related to screening and diagnosis of neonatal hyperbilirubinemia and 3 were related to treatment. On the basis of these indicators, we established predefined criteria of best practices in management of neonatal hyperbilirubinemia and applied this protocol to our medical record review. On the basis of compliance with these best practices and using the classification of the National Board of Health and Welfare in Sweden,^21^ we characterized cases of kernicterus as unavoidable, potentially unavoidable, potentially avoidable (defined as a predominant probability ≥51% for avoidable injury), or avoidable (defined as a predominant probability ≥75% for avoidable injury) given the best available knowledge had been practiced. This classification is mentioned in the Swedish Patient Safety Act (chapter 2, section 6), and the probability cutoffs chosen herein are used by medical advisors to the Swedish Patient Insurance (lower cutoff) and in legal practice (higher cutoff) when judging claims from families.

Finally, we defined the proportion of kernicterus cases for which financial compensation had been applied for and had been granted from the Swedish Patient Insurance.

### Statistical Analysis

Data analysis was performed between September 2017 and February 2018. Data are presented as number and proportions (percentages or rates per 100 000 live births), mean (SD or 95% CIs,) or as median (range) values. We used *t* tests to compare maximum bilirubin levels between infants with hazardous hyperbilirubinemia who developed kernicterus and those who did not. A 2-sided *P* < .05 was considered statistically significant. Analyses were conducted using IBM SPSS, version 23 (SPSS Inc).

## Results

In the study period, there were 992 378 live-born infants, of whom 958 051 were born at term and 34 327 were born at 35 to 36 weeks of gestational age. Among these, 494 infants (320 boys; mean [SD] birth weight, 3505 [527] g) were registered in the SNQ with extreme hyperbilirubinemia (serum bilirubin level, 25.0-29.9 mg/dL or 425-509 μmol/L), corresponding to an incidence rate of 50 per 100 000 infants. In 67 infants (49 boys, mean [SD] birth weight, 3540 [547] g), the maximum serum bilirubin level was 30 mg/dL (510 μmol/L) or higher, corresponding to an incidence rate of 6.8 per 100 000 infants. Selected maternal and infant characteristics of those with neonatal serum bilirubin levels of 30 mg/dL (510 μmol/L) or higher are presented in Table 1. Despite newly issued national guidelines at the start of the study period, the annual rate of infants with hazardous hyperbilirubinemia appeared to increase during the study period (Figure). The highest serum bilirubin level registered was 50 mg/dL (847 μmol/L).

**Table 1.** Perinatal Characteristics of Newborns (≥35 Weeks’ Gestational Age) With Serum Bilirubin Levels of 30 mg/dL or Higher^a^

Characteristic	Newborns, No. (%)
Total No.	67
Family history	
Of hemolytic disease	3 (4)
Sibling treated for neonatal hyperbilirubinemia	8 (12)
Maternal characteristic	
Primipara	24 (36)
Asian	14 (21)
Overweight or obese	16 (24)
Diabetes, any	0
Blood group O or Rh negative	40 (67)
Mode of delivery	
Vaginal, noninstrumental	60 (90)
Vaginal, vacuum extraction	3 (4)
Cesarean	4 (6)
Gestational age, wk	
35-36	4 (6)
37-38	38 (57)
39-40	19 (28)
≥41	6 (9)
Infant characteristic	
Male sex	49 (73)
Birth weight, median (range), g	3542 (2290-4600)
SGA	1 (1)
AGA	55 (82)
LGA	11 (16)
Maximum serum bilirubin level, median (range), mg/dL	32 (30-50)
Maximum serum bilirubin level, median (range), μmol/L	542 (510-847)

Abbreviations: AGA, appropriate for gestational age; LGA, large for gestational age; SGA, small for gestational age.

^a^ The SI unit for the bilirubin levels is 510 μmol/L or higher.

**Figure.**
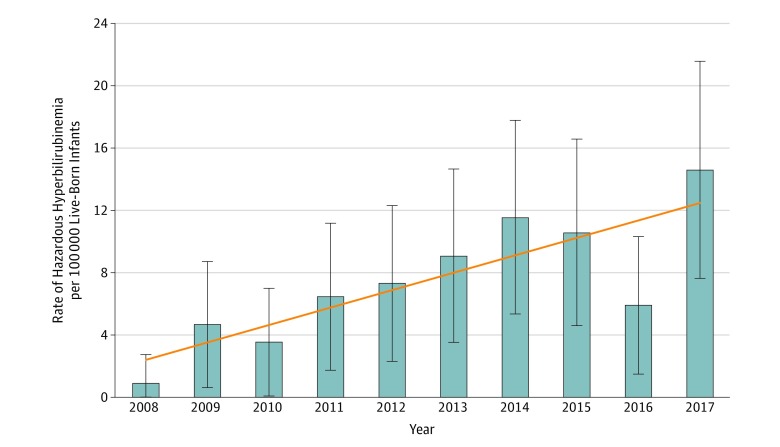
Annual Rate of Newborns (≥35 Weeks’ Gestational Age) With Hazardous Serum Bilirubin Levels (≥30 mg/dL) in Sweden Error bars indicate 95% CIs; diagonal line, linear trend over time (the SI unit for the bilirubin levels is ≥510 μmol/L).

### Risk Factor Profiles

Among infants with hazardous hyperbilirubinemia, the most common risk factors were male sex (49 of 67 [73%]), maternal blood group O or Rh negative (40 of 67 [60%]), or gestational age 35 to 38 weeks (42 of 67 [63%]). The median (range) number of risk factors for hyperbilirubinemia in each infant was 4 (1-10).

In Sweden, 1.9% of all term infants are treated for nonhemolytic hyperbilirubinemia, and 0.6% are treated for hemolytic hyperbilirubinemia.^3,4^ Adopting a previously developed risk-score algorithm for nonhemolytic neonatal hyperbilirubinemia based on a few maternal and obstetric risk factors,^4^ 29 of 67 infants (43%) could have been classified at birth as being at high (>10%) to very high (5%-10%) risk, 28 of 67 infants (42%) at moderate risk (>1% but <5%), and 10 of 67 infants (15%) at low risk (<1%) at birth of developing hyperbilirubinemia in need of treatment.

### Underlying Diagnoses and Conditions

Hemolytic disease was diagnosed in 33 of 67 infants (49%) with hazardous hyperbilirubinemia (serum bilirubin level ≥30 mg/dL [≥510 μmol/L]). The most common underlying diagnosis was ABO isoimmunization, whereas no infant exposed to hazardous hyperbilirubinemia could be ascribed to Rh isoimmunization. All cases with ABO isoimmunization had a positive Coombs test result. Documented hematoma was the most common condition among infants with nonhemolytic hyperbilirubinemia. In 18 of 67 cases of hazardous hyperbilirubinemia (27%), no underlying disease or contributing medical factor could be found during the record review (Table 2).

**Table 2.** Associated Diagnoses and Conditions Among Newborns (≥35 Weeks’ Gestational Age) With Serum Bilirubin Levels of 30 mg/dL or Higher^a^

Diagnosis or Condition	Newborns, No. (%)
Total No.	67
Hemolytic disease	33 (49)
ABO blood type isoimmunization	23 (34)
G6PD deficiency	8 (12)
Rh isoimmunization	0
Other (spherocytosis)	2 (3)
Nonhemolytic origin	16 (24)^b^
Hematoma, bleeding	13 (19)
Polycythemia	3 (4)
Preterm birth	2 (3)
No identifiable underlying or contributing disease or risk factor	18 (27)

Abbreviation: G6PD, glucose-6-phosphate dehydrogenase.

^a^ The SI unit for the bilirubin levels is 510 μmol/L or higher.

^b^ Two infants with more than 1 diagnosis.

### Bilirubin Encephalopathy and Kernicterus

Among infants with serum bilirubin levels of 30 mg/dL (510 μmol/L) or higher, 28 of 67 (42%) had 1 or more symptoms of acute bilirubin encephalopathy documented in their medical records. Thirteen infants—all with signs of acute encephalopathy and with serum bilirubin levels between 30 and 45 mg/dL (517-765 μmol/L)—developed kernicterus (incidence rate, 1.3; 95% CI, 0.6-2.0 per 100 000). The maximum bilirubin level among those who developed kernicterus was higher than that in infants who did not develop kernicterus (mean [SD], 36.0 [4.6] mg/dL or 608 [79] μmol/L vs 32.0 [3.2] mg/dL or 551 [54] μmol/L; *P* = .01).

### Adherence to Best Practices

By auditing the medical records of 67 infants with hazardous bilirubin levels using our predefined performance indicators, we found that large proportions of infants had been discharged early from the hospital without a predischarge bilirubin level determination (eg, for 15 of 19 infants [79%] leaving the hospital <24 hours after birth) and that untimely or no exchange transfusion was common (eg, >12-hour delay or no exchange for 19 of 28 infants [68%]) (Table 3).

**Table 3.** Nonadherence to Key Performance Indicators for Newborns (≥35 Weeks’ Gestational Age) With Serum Bilirubin Levels of 30 mg/dL or Higher^a^

Screening and Diagnosis	Newborns, No./Total No. (%)
Bilirubin level not determined before discharge in infants leaving the hospital <24 h after birth	15/19 (79)
Bilirubin level not determined before discharge in infants leaving the hospital <48 h after birth (includes infants leaving hospital <24 h after birth)	28/41 (68)
Hospital discharge without an appointed time for repeated bilirubin level determination despite a bilirubin level between the 70th and 95th centile^5^	15/62 (24)
Serum bilirubin level not determined although the first transcutaneous bilirubin level was >15 mg/dL (250 μmol/L)	3/5 (60)
Bilirubin testing not repeated within 24 h despite an increase >100 μmol/L in 24 h	9/17 (53)
Bilirubin testing not repeated within 24 h despite a bilirubin value between the 70th and 95th centile^5^	5/13 (38)
Outpatient clinic follow-up not scheduled despite hospital discharge <48 h after birth	7/56 (12)
Substantial jaundice at follow-up without determination of bilirubin level	2/51 (4)
**Treatment**^b^	
Untimely (>6-h physician delay) administration of high-intensity phototherapy	5/67 (7)
Untimely (>12-h physician delay) or no exchange transfusion	19/28 (68)
Repeated exchange transfusion not performed when indicated (serum bilirubin level after exchange exceeded recommended limit for exchange transfusion)	2/4 (50)

^a^ Based on Swedish national guidelines for screening and diagnosis of neonatal hyperbilirubinemia issued in 2008^20^ (the SI unit for the bilirubin levels is ≥510 μmol/L).

^b^ Time intervals set by the authors.

### Avoidance of Kernicterus

Among 13 children with a final diagnosis of kernicterus, brain injury was judged as potentially avoidable for 6 and avoidable for 5 based on 1 or several of the following criteria: untimely or lack of bilirubin level screening (n = 6), misinterpretation of bilirubin values (n = 2), untimely or delayed initiation of treatment with intensive phototherapy (n = 1), untimely or delayed or no treatment with exchange transfusion (n = 6), or lack of repeated exchange transfusions despite indication (n = 1).

Kernicterus was assessed as potentially unavoidable in only 1 infant (a slightly preterm infant with trisomy 21) and as unavoidable in only 1 infant (a term newborn without any risk factors who was screened and followed up according to national standards but was readmitted from home with extreme hyperbilirubinemia and eventually diagnosed as having G6PD deficiency).

### Financial Compensation

During the study period, 6 of 13 families affected by kernicterus had applied for and had been granted financial compensation from the Swedish National Patient Insurance. Besides those infants, all of whom were also included in the SNQ register, 1 additional infant who had received a diagnosis of neonatal hyperbilirubinemia was found in the database of the Swedish National Patient Insurance.

## Discussion

The 3 most clinically relevant and important observations of this study were that (1) hazardous hyperbilirubinemia and kernicterus among near-term to term newborns is still occurring and possibly even increasing in Sweden; (2) almost half of those newborns developing hazardous bilirubin levels could have been identified, without a blood test, as being at high to very high risk for hyperbilirubinemia already at birth; and (3) among those developing kernicterus, 85% of the cases were most likely avoidable because they were associated with nonadherence to best practices and substandard care.

The incidence rate of kernicterus (1.3 per 100 000 births) found in the present study appears to be slightly higher than that in other population-based studies in high-resource settings. In Canada, California, and Denmark, the incidence rate of kernicterus has been reported to be 0.5 to 1 per 100 000 births,^13,15,17,22,23,24^ whereas in Norway, the incidence rate has been estimated to be less than 0.5 per 100 000 births.^25^ The incidence rate of hazardous neonatal hyperbilirubinemia (≥30 mg/dL or ≥510 μmol/L) was 6.8 per 100 000 births in our study, which is in line with reports from other European countries and the United States, varying from 7 to 45 per 100 000 births.^16,26,27,28^ Much higher rates (1500-3000 per 100 000 births) have been observed in Asian low- to middle-income countries.^12,29,30^

On the basis of a few and easily available maternal and obstetric risk factors,^4^ high to very high risk of hyperbilirubinemia requiring treatment could have been anticipated at birth in almost half the newborns with hazardous bilirubin levels. Although this might be clinically useful information, collection of additional data on specificity will be needed before the true predictive value of this risk factor tool can be calculated. In addition, the severity of hyperbilirubinemia cannot be determined by results of high to very high risk using this algorithm; such risk outcomes merely offer an early warning that could be useful in planning for timing of bilirubin testing and follow-up.

As expected, a diagnosis of hemolytic disease (ABO isoimmunization or G6PD deficiency) was found to be a major contributing factor of hazardous hyperbilirubinemia in the present study. The finding that 1 in 4 infants developing hazardous hyperbilirubinemia had no underlying disease or condition was more surprising and illustrates that apparently heathy babies may also be affected.^31,32^ Our finding could also signal flaws in investigation of underlying causes.

In the present study, 1 in 5 infants with hazardous hyperbilirubinemia developed kernicterus, and more than half of these cases were associated with lack of screening and diagnosis. We can only speculate about the explanations for these adverse events, such as unawareness of the national guidelines or specific items within them, nonacceptance of the recommendations based on limitations in the evidence, or restraints of time and limited staff resources. Perhaps also important, the digital records in Swedish maternity units include only limits for phototherapy and exchange transfusion in the hour-specific bilirubin medical records for newborns, withholding risk zones and decision aids on monitoring and follow-up at different bilirubin levels that are provided in the national guidelines from 2008. Digital safeguards, such as those used in other countries, could be one way forward, especially if the safeguards are integrated into the existing medical record system and are made available to parents.

Another mismanagement of hazardous hyperbilirubinemia that our study highlighted was untimely or even no exchange transfusion despite serum bilirubin levels of 30 mg/dL (510 μmol/L) or higher and symptoms of acute bilirubin encephalopathy. Since the introduction of a successful program for prevention and antenatal treatment of Rh hemolytic disease, exchange transfusions have been less frequently performed in Sweden (mean national rate from 2012 to 2016, 0.4 exchange transfusions per 1000 births). Many infants also respond very effectively to intensive phototherapy.^33^ Both factors may have contributed to the development of a less active and reluctant approach toward exchange transfusions.

In Sweden, patients who experience an injury while in the health care system may in some cases be entitled to financial compensation under the Patient Injury Act. Approximately 16 000 injuries are reported to the Patient Injury Insurance annually, and 40% of them are compensated. Avoidable brain damage in the neonatal period is one of the highest compensated conditions. All 6 families in our study who applied for compensation were granted financial compensation, whereas 5 families with a child having kernicterus that was deemed avoidable by the present study never applied for compensation. This could reflect unawareness among families despite health care professionals being obliged to inform patients and parents about the patient insurance in cases of patient injury. It may also reflect a reluctance among physicians to acknowledge kernicterus as a patient injury.

### Strengths and Limitations

The strengths of the present study include the large population base, use of information from different registers and databases, validation of outcome data against medical records, and evaluation of best practices using a predefined protocol based on national guidelines. We included all infants born in Sweden within a decade who developed hazardous hyperbilirubinemia, collected their risk factor profiles and diagnoses, and scrutinized their medical records up to 2 years of age, enabling us to present true population-based incidence rates of kernicterus and its causes. Including not only the diagnoses and interventions in our quality register but also the maximum serum bilirubin levels has built a national surveillance system with potential to critically evaluate practice at the national, regional, and individual family levels.

Our study also had some limitations. It was retrospective in design and may therefore lack some information. We had information on only maximum serum bilirubin levels from the SNQ, and we did not collect more bilirubin values because they were not uniformly assessed or documented in medical records. We did not have a control group because this study was never intended to compare characteristics or risk factors among infants with kernicterus with those of the general population. Risk factors and characteristics of term and near-term infants developing significant hyperbilirubinemia in our population have been previously reported.^4^ In addition, the structured medical record reviews were limited to worst-case scenarios and offer little information about clinical performance regarding the general management of hyperbilirubinemia. However, considering that outcomes, such as kernicterus, most likely represent the tip of the iceberg, we have no reason to believe that noncompliance with best practice guidelines was isolated to the infants studied herein.

The explanation for the apparent annual increase in extreme hyperbilirubinemia was unclear. It could be attributable to improved tracking in later years. Considering that the national guidelines from 2008 onward recommended more frequent bilirubin testing, this recommendation could have contributed to an increase in detection rates over time. If so, the actual incidence rates could have been underestimated, at least in the beginning of the study period. In the first study year (2008), underreporting to the register may have been a problem based on our general observation of the annual rates of reported phototherapy for treatment of hyperbilirubinemia. We have no knowledge of changes in bilirubin measurement techniques during the study period. An accelerated shortage of specialist nurses and a decrease in the number of midwives have contributed to reduced systems capacity to a level at which patient safety might have been affected. The results of the present study indicated that unrecognition of risk factors and unawareness of recommendations in guidelines as well as early hospital discharge without appropriate follow-up appeared to be the most significant problems.

The Swedish MBR includes information only about the neonatal period; therefore, it is not surprising that infants coded with kernicterus were misclassified. To avoid such misclassification in the future, we suggest that in the next revision of *ICD* codes, kernicterus should be excluded from the perinatal section. Although kernicterus is a condition originating in the perinatal period, it cannot be diagnosed until after the perinatal period and therefore should instead be included in the *ICD* sections covering pediatric or neurologic conditions.

## Conclusions

In summary, among 100 000 near-term to term live-born infants in Sweden, 6.8 developed hazardous hyperbilirubinemia and 1.3 developed kernicterus. Among infants who developed brain damage associated with hazardous hyperbilirubinemia, 85% were deemed avoidable by the criteria used in the present study. The challenge and the way forward to prevent kernicterus include not only evidence-based guidelines advocating bilirubin level determination before hospital discharge but also improved digital solutions and automated safeguards for decision making regarding follow-up, diagnosis, and treatment. Finally, seeking explanations for cases of hazardous hyperbilirubinemia and kernicterus in which no underlying cause can be found should be given priority.^17,34^

## Notes

Supplement. **eFigure.** Bilirubin chart used in Sweden as a basis for decision-making in near-term and term infants
