# Supplementary material for: Rates of Extreme Neonatal Hyperbilirubinemia and Kernicterus in Children and Adherence to National Guidelines for Screening, Diagnosis, and Treatment in Sweden
Source: JAMA Netw Open. 2019 Mar 22;2(3):e190858. doi: 10.1001/jamanetworkopen.2019.0858 (PMC6583272; doi:10.1001/jamanetworkopen.2019.0858)
Supplement: Supplement. — eFigure. Bilirubin chart used in Sweden as a basis for decision-making in near-term and term infants [file jamanetwopen-2-e190858-s001.pdf]

Supplementary Online Content

Alkén J, Håkansson S, Ekéus C, Gustafson P, Norman M. Rates of extreme neonatal hyperbilirubinemia and kernicterus in children and adherence to national guidelines for screening, diagnosis, and treatment in Sweden. *JAMA Netw Open*. 2019;2(3):e190858. doi:10.1001/jamanetworkopen.2019.0858

eFigure. Bilirubin chart used in Sweden as a basis for decision-making in near-term and term infants.

This supplementary material has been provided by the authors to give readers additional information about their work.

© 2019 Alkén J et al. *JAMA Network Open*.

eFigure. Bilirubin chart used in Sweden as a basis for decision-making in near-term and term infants.

Bilirubin chart

Newborns ≥35 wks GA

Suggested treatment limits		
GA, weeks+days	Phototherapy	X-change transfusion
35+0 – 36+6	300	380
≥37+0	350	425

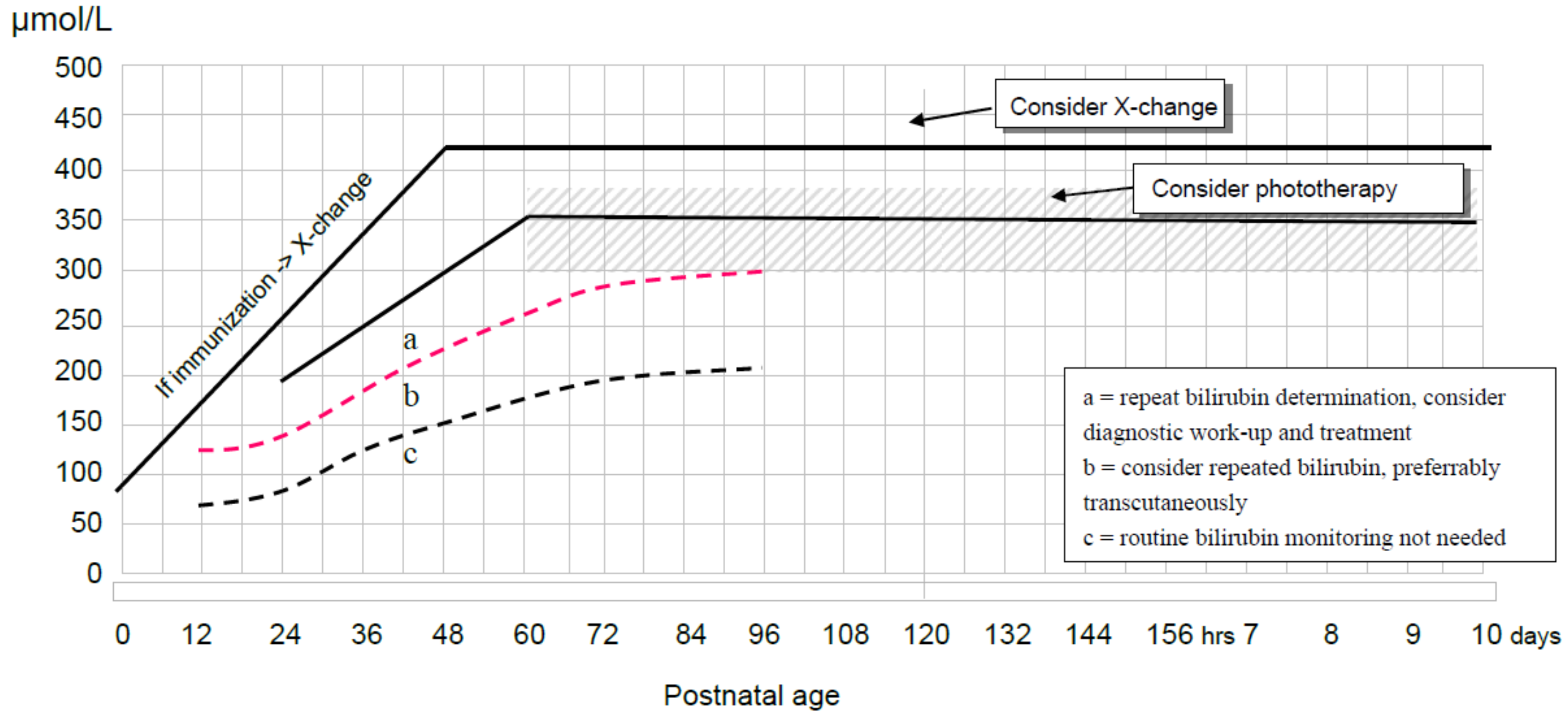

© 2019 Alkén J et al. *JAMA Network Open*.
